# Feasibility of fNIRS in Children with Developmental Coordination Disorder

**DOI:** 10.1192/j.eurpsy.2022.179

**Published:** 2022-09-01

**Authors:** C. Johnson, K. Klingels, E. Verbecque, P. Meyns, A. Hallemans

**Affiliations:** 1 University of Antwerp; Hasselt University, Rehabilitation Sciences And Physiotherapy; Research Group Movant; Reval, Wilrijk (Antwerp), Belgium; 2 Hasselt University, Rehabilitation Sciences And Physiotherapy; Research Group Reval, Diepenbeek, Belgium; 3 University of Antwerp, Research Group Movant, Department Rehabilitation Sciences And Physiotherapy, Wilrijk, Belgium

**Keywords:** Developmental Coordination Disorder, brain imaging, Pediatric, movement science

## Abstract

**Introduction:**

Balance deficits are heterogeneous among children with Developmental Coordination Disorder (DCD). Balance performance depends on different balance domains, each associated with specific underlying neurological systems. In DCD, any of these domains can be affected, but the control mechanisms are poorly understood. The mirror neuron system (MNS) seems to play a key-role in DCD-related deficits. To understand the role of MNS as a control mechanism underlying the balance deficits, simultaneous registration of cortical MNS activity while performing balance tasks is imperative. Therefore, a protocol for combining real-time registration of cortical MNS activity during functional balance tasks in children with DCD, CP and TD is introduced. Methods: Children with DCD, CP and TD (n=108) aged 5-10yr perform preselected tasks of the Kids-BESTest, representing specific balance domains (mixed design): leaning with eyes closed (stability limits/verticality), single-leg-stance, alternate stair touching (anticipatory balance), in-place response, compensatory stepping backward (reactive balance) and walking over obstacles (gait stability). Simultaneously, functional Near-Infrared Spectroscopy (fNIRS) monitors cortical activity involving the MNS: premotor, inferior and superior parietal cortex and supplementary motor area. An 8-8-optode bundle, making 22 channels, targets this region of interest. Outcome measures are: (de)oxygenated hemoglobin concentration changes per task per channel. Results: In this ongoing research, the protocol was already feasible in 19 children (7.52±1.19). Conclusion: Simultaneous registration of cortical MNS activity (fNIRS) and Kids-BESTest scores will help increase the understanding of the control mechanisms underlying the heterogeneous balance problems in DCD. Consequently, first steps are made to confirm whether DCD shows deviant or delayed development.

**Disclosure:**

No significant relationships.

